# The association between physical activity, sedentary behavior, sleep, and body mass index z-scores in different settings among toddlers and preschoolers

**DOI:** 10.1186/s12887-016-0642-6

**Published:** 2016-07-20

**Authors:** Nicholas Kuzik, Valerie Carson

**Affiliations:** Faculty of Physical Education and Recreation, University of Alberta, Edmonton, AB T6G 2H9 Canada

**Keywords:** Physical activity, Sedentary behavior, Sleep, Body mass index, Children, Toddler, Preschooler, Child care

## Abstract

**Background:**

Physical activity, sedentary behavior, and sleep are all movement behaviors that range on a continuum from no or low movement, to high movement. Consistent associations between movement behaviors and adiposity indicators have been observed in school-age children. However, limited information exists in younger children. Since approximately 50 % of Canadian children ≤5 years of age attend non-parental care, movement behaviors within and outside of the child care setting are important to consider. Therefore, this study examined the association between movement behaviors (physical activity, sedentary behavior and sleep) inside and outside of child care, with body mass index (BMI) z-scores, among a sample of toddlers and preschoolers.

**Methods:**

Children aged 19–60 months (*n* = 100) from eight participating child care centers throughout Alberta, Canada participated. Movement behaviors inside child care were accelerometer-derived (light physical activity, moderate to vigorous physical activity (MVPA), sedentary time, and time spent in sedentary bouts lasting 1–4, 5–9, 10–14 and ≥15 min) and questionnaire-derived (daytime sleep). Movement behaviors outside of child care were questionnaire-derived (MVPA, screen and non-screen sedentary behavior, and nighttime sleep). Demographic information (child age, child sex, and parental education) was also questionnaire-derived. Height and weight were measured, and age- and sex-specific BMI z-scores were calculated using World Health Organization growth standards. The association between movement behaviors and BMI z-scores were examined using linear regression models.

**Results:**

Hours/day of sedentary bouts lasting 1–4 min (β =−0.8, 95 % CI:−1.5,−0.1) and nighttime sleep (β = 0.2, 95 % CI: 0.1, 0.4) were associated with BMI z-scores. However, after adjusting for demographics variables, sedentary bouts lasting 1–4 min (β =−0.7; 95 % CI:−1.5, 0.0) became borderline non-significant, while nighttime sleep (β = 0.2, 95 % CI: 0.1, 0.4) remained significant. No other movement behaviors inside/outside of child care were associated with BMI z-scores.

**Conclusions:**

All children must engage in some sedentary behavior in a day, but promoting the sedentary behavior in short bouts during child care may be important for the primary prevention of overweight and obesity. Future research is needed to understand the mechanisms between sleep and adiposity in this age group and to confirm these findings in large representative samples.

## Background

The intensity of movement can range on a continuum from minimal-intensity during sleep or sedentary behavior, to high-intensity during vigorous physical activity [1]. Consistent positive associations between low physical activity, excess sedentary behavior, and insufficient sleep and adiposity have been reported in school-aged children and youth (aged 5–17 years) [2–4]. However, less is known regarding the associations between movement behaviors (i.e., physical activity, sedentary behavior, and sleep) and adiposity measures in early years children (aged 0–5 years). Movement behavior habits formed in the early years have implications for future health [5]. Therefore, understanding the associations between these behaviors and adiposity indicators in this age group, could inform health promoting initiatives.

Only one study has examined the association between the full spectrum of movement behaviors and adiposity in the early years. This longitudinal study found a negative association between accelerometer-derived sleep at ages 3–5, and body mass index (BMI) at age 7 in a sample of 244 children [6]. However, accelerometer-derived total physical activity and parent reported TV viewing at ages 3–5, showed no associations with BMI at age 7 [6].

Several gaps in the literature exist regarding the associations between the full spectrum of movement behaviors and adiposity in the early years. First, no study has examined these associations in different settings. The importance of the home setting for children of the early years is well known [7–9]. Another important setting for early years children is child care. In Canada, 54 % of children accumulate a large proportion (29 h/week) of their time inside child care centers [10]. One study found, attending child care was associated with an increased risk of overweight/obesity, compared to parental care [11]. Thus, it is important to examine associations between movement behaviors and adiposity both inside and outside of child care, to determine if setting specific initiatives are needed for the health of early years children. Second, there is limited research on the association between movement behaviors and adiposity in children <3 years, which makes it unclear whether there is an optimal age to intervene to prevent overweight and obesity. Finally, it is unknown whether specific demographic groups (e.g., girls, children with educated parents) are at an increased risk and therefore should be specifically targeted for intervention.

The primary purpose of this study was to examine the associations between the full spectrum of movement behaviors (i.e., physical activity, sedentary behavior, and sleep) and BMI z-scores, inside and outside of child care centers, in a sample of 19–60 month olds. The secondary purpose of this analysis, and was to examine if the associations between movement behaviors and BMI z-scores were moderated by age, sex, and parental education.

## Methods

### Participants

The Supporting Active Living Behaviors in Alberta Child Care Settings study examined the effects of revised Child Care Accreditation Program Quality Standards in Alberta, Canada, as described in full detail elsewhere [12]. Child care centers in Alberta scheduled for initial accreditation during August to October 2013 were eligible for the study. Out of the 12 eligible centers, eight (67 %) agreed to participate. Seven centers were located in the cities of Edmonton (*n* = 4) and Calgary (*n* = 3), while one center was located in a smaller city in Alberta. The current study presents cross-sectional baseline data from the full quasi-experimental study [12].

Parents with a child aged 19 to 60 months who typically attended participating child care centers full-time (i.e., >4 h/day) were invited to participate. Out of the 270 eligible children, 145 (54 %) had a parent return a signed consent form and questionnaire. Four children were excluded based on age restrictions, leaving 141 children. Data were collected between September and November 2013. The University of Alberta Health Research Ethics Board provided ethics approval for this project. Written and informed consent was provided by the parents

### Adiposity measure

Children’s weight was measured twice to the nearest 0.1 kg using a digital scale, and height was measured twice to the nearest 0.1 cm using a stadiometer. If a difference of >0.2 units were scored between the two measurements of height or weight, a third measurement was performed and the average of the two closest measurements were used. BMI z-scores were calculated according to the World Health Organization (WHO) growth standards [13]. BMI z-scores are defined as normal-weight (−1.99–0.99), at risk of becoming overweight (1.0–1.9), and overweight (≥2) [14].

### Movement behaviors inside child care

#### Physical activity and sedentary behavior

Accelerometers (Actical, Respironics, Bend, OR, USA), calibrated for the study, were fitted over the right hip with a belt by research staff. After this initial fitting, on the first morning of data collection, early childhood educators attached and removed the belts daily as children arrived and left the centers. Children continuously wore the accelerometer during five consecutive weekdays. Accelerometer data were collected in 15-s epochs [15]. Non-wear time was defined as sequences of consecutive zero counts ≥20 min and was excluded from analyses [16]. Early childhood educators filled out log-sheets indicating children’s accelerometer on and off times. Log-sheets were used to cross-reference non-wear time and to remove data points prior to the start time of the first data collection day. Daytime sleep was assumed to be excluded with the non-wear time definition. This was confirmed by cross-referencing log-sheet data, where daytime sleep was recorded. Since some centers did not record daytime sleep on the log-sheets, daytime sleep during child care was measured with parental questionnaires. This was based on the assumption that parents would know children’s napping habits through conversations with child care educators and weekend observations. Consistent with previous studies inside child care centers [17, 18], participants with ≥1 h of wear time on ≥3 days were considered to have valid data and therefore included in the analyses.

Based on national survey data from the Canadian Health Measures Survey [19], accelerometer cut-points were defined as: sedentary behavior (<100 cpm; cpm or <25 counts/15 s), light-intensity physical activity (LPA; 100 to 1149 cpm or 25 to <287.5 counts/15 s), and moderate- to vigorous-intensity physical activity (MVPA; ≥1150 cpm or ≥287.5 counts/15 s). This MVPA cut-point has significantly greater classification accuracy compared to other Actical accelerometer cut-points in this age group [20]. Sedentary behavior, LPA, and MVPA were expressed as hours/day. Time spent in sedentary bouts (hours/day) lasting 1–4, 5–9, 10–14, and ≥15 min were also calculated, while allowing for zero tolerance of interruption [21]. SAS version 9.4 [SAS Institute Inc., Cary, NC] was used for accelerometer data reduction. Consistent with previous studies [22, 23], accelerometer variables were adjusted for wear time by standardizing the variables using the residuals obtained with regressing the variables on the corresponding wear time variable [24].

#### Sleep

Daytime sleep during child care was determined by asking parents: *“How long does your child usually nap during the day at the moment?”* Responses for hours and minutes were used to calculate an hours/day variable.

### Movement behaviors outside of child care

#### Physical activity

MVPA was assessed by asking parents two questions: “*About how many hours a week does your child usually take part in physical activity (that makes him/her out of breath or warmer than usual) outside of child care while participating in…*” both *“…organized activities (*e.g.*, swimming lessons, skating lessons, gymnastics)?”* and *“…non-organized activities (*e.g.*, going for a walk, drop-in skating, playing at a splash pad or wading pool, bike or tricycle ride, playing at the park or in the yard)?”* For both questions, a 5-point scale ranging from *“never”* to *“7+ hours/week”* was used. Consistent with previous research, the mid-points, unless starting/end point, of the responses in hours (i.e., 0.0, 1.0, 2.5, 5.0, and 7.0 h) were calculated and the values for both questions were then summed and converted to an hours/day variable [19]. Questions were adopted from Statistic Canada’s Canadian Health Measures Survey [19].

#### Sedentary behavior

Screen based sedentary behavior was assessed by asking parents two questions: *“On average, how much time per day outside of child care does your child…”* both *“…watch television, videos or DVDs on a television, computer or portable device?”* and *“…play video/computer games on devices such as a learning laptop, leapfrog leapster, computer, laptop, tablet, cell phone, the internet, Playstation, XBOX?”*. Non-screen time sedentary behavior was assessed by asking parents two questions: *“On average, how much time per day outside of child care does your child spend…”* both *“…in a motor vehicle (*e.g.*, car, LRT [light rail transit], bus)?”* and *“…being safely restrained in a high chair, stroller,* etc. *(do not include when they are in a motor vehicle)?”* For all questions, a 7-point scale ranging from *“none”* to *“3 h or more”* were used for weekdays and weekend days separately. Consistent with previous research, the mid-points, unless start/end points, of the responses in minutes (i.e. 0.0, 7.5, 22.5, 45.0, 90.0, 150.0, and 180.0 min) were calculated and then converted to hours [19]. Weighted means (weekday mean × 5 + weekday mean × 2 /7) were calculated to create hour/day variables for both screen time sedentary behavior and non-screen time sedentary behavior. The screen based sedentary behavior questions were adopted from the Statistic Canada’s Canadian Health Measures Survey [19].

#### Sleep

Total nighttime sleep was assessed by asking parents: *“How long does your child usually sleep per night at the moment?”* Responses for hours and minutes were used to calculate an hours/night variable.

### Covariates

Age (in months), sex (male or female), and parental education (grade 1–8, grade 9–12, community/technical college, university, or graduate university) were assessed in the parental questionnaire.

### Statistical analysis

SPSS version 22.0 [IBM Corp., Armonk, NY] was used to perform statistical analyses. Descriptive statistics were calculated including means, standard deviations, and percentages. Movement behavior variables deemed outlier’s (≥ ±3 standard deviations) were truncated to the nearest non-outlier value (*n* = 3). To assess if a multilevel regression model was needed to account for variance in BMI z-scores between child care centers, a null model was created to determine the intra-class correlation coefficient [25]. An intra-class correlation coefficient of 0.004 indicated a multilevel regression model was not necessary [25]. Therefore, simple linear regression models were first conducted between movement behaviors and BMI-z scores (model 1). Multiple linear regression models were also conducted that adjusted for age, sex, and parental education (model 2). Lastly, age, sex, and parental education moderating effects were tested by including interaction terms in the model one at a time. Statistical significance was set at *P* < 0.05 for all analyses.

## Results

Out of the 141 participants that agreed to participate, 100 children had complete data for the variables of interest. Age, sex, and BMI z-scores did not significantly differ between included and excluded participants. However, excluded participants on average had less educated parents. Participant characteristics are presented in Table 1. Children in the final sample were on average 38.5 (standard deviation (SD) = 12.1) months of age, 47.0 % were female, and 49.0 % had a parent with a University education below graduate school. Children were classified as overweight (*n* = 3), at risk for overweight (*n* = 25), and normal weight (*n* = 72). Inside child care, children’s average total accelerometer wear time was 5.7 (SD = 1.5) hours/day, which was predominantly spent sedentary (3.4 h/day sedentary / 5.7 h total wear time = 59.6 % of time). Furthermore, most sedentary time was spent in 1–4 min bouts (1.3 h/day in 1–4 min bouts/ 3.4 h sedentary = 38.2 %), compared to other sedentary bout lengths. Children accumulated 1.2 (SD = 0.9) hours/day of daytime sleep, and 10.3 (SD = 1.0) hours/day of nighttime sleep. During waking hours, screen time was the most prevalent movement behavior outside of child care accounting for 2.1 (SD = 1.1) hours/day.

**Table 1:** Participant characteristics

Variables	Total (*n* = 100)
Adiposity measure	
BMI z-score^a^	0.5 (0.8)
Movement behaviors inside child care	
Number of valid weekdays^b^	4.6 (0.8)
Accelerometer wear time (hours/day)^b^	5.7 (1.5)
LPA (hours/day)^b^	1.8 (0.3)
MVPA (hours/day)^b^	0.5 (0.3)
Sedentary time (hours/day)^b^	3.4 (0.5)
Sedentary bouts (hours/day) lasting	
1–4 min^b^	1.3 (0.2)
5–9 min^b^	0.6 (0.2)
10–14 min^b^	0.4 (0.2)
≥ 15 min^b^	0.5 (0.3)
Daytime Sleep (hours/day)^c^	1.2 (0.9)
Movement behaviors outside of child care	
MVPA (hours/day)^c^	1.0 (0.4)
Screen time (hours/day)^c^	2.1 (1.1)
Non-screen time (hours/day)^c^	1.8 (0.9)
Nighttime sleep (hours/night)^c^	10.3 (1.0)
Covariates	
Age (months)^c^	38.5 (12.1)
Sex	
Female^c^	47.0 %
Male^c^	53.0 %
Parental Education	
Grade 9–12^c^	11.0 %
Community/Technical College^c^	22.0 %
University (e.g., undergraduate, teacher’s college)^c^	49.0 %
Graduate University (e.g., master’s, doctorate, medicine)^c^	18.0 %

Values represent mean (standard deviation) for continuous variables and percentage for categorical variables; *BMI* body mass index, *LPA* light physical activity, *MVPA* moderate-to-vigorous physical activity

^a^Researcher measured variable; ^b^Accelerometer measured variable; and ^c^Parental-questionnaire measured variable

Results for the regression models are presented in Table 2. Time spent in sedentary bouts (hours/day) lasting 1–4 min (Unstandardized regression coefficient (β) =−0.8; 95 % confidence interval (95 % CI):−1.5,−0.1) was significantly associated with BMI z-scores in model 1. However, no other movement behaviours within child care were associated with BMI z-scores. Outside of child care, only nighttime sleep (hours/night) (β = 0.2; 95 % CI: 0.1, 0.4) was significantly associated with BMI z-score in model 1. After controlling for age, sex, and parental education in model 2, only nighttime sleep (hours/night) (β = 0.2; 95 % CI: 0.1, 0.4) remained significant (*P* = 0.01). However, within child care, time spent in sedentary bouts (hours/day) lasting 1–4 min (β =−0.7; 95 % CI:−1.5, 0.0) was borderline non-significant (*P* = 0.05) in the adjusted model. Beyond sedentary bouts lasting 1–4 min and nighttime sleep, no other movement behaviours were significantly associated with BMI z-score in either model. Further, no significant age, sex, and parental education interactions were observed for any of the movement variables.

**Table 2:** Associations between movement behaviors and BMI z-scores inside and outside of child care

	Model 1	Model 2
	β (95 % CI)	β (95 % CI)
Movement behaviors inside child care		
Physical activity		
LPA (hours/day)	0.15 (−0.36, 0.66)	0.31 (−0.22, 0.84)
MVPA (hours/day)	0.11 (−0.52, 0.75)	0.41 (−0.26, 1.08)
Sedentary behavior		
Sedentary time (hours/day)	−0.10 (−0.45, 0.24)	−0.27 (−0.64, 0.09)
Sedentary bouts (hours/day) lasting		
1–4 min	−0.80 (−1.53, -0.07)*	−0.73 (−1.47, 0.01)
5–9 min	−0.16 (−0.93, 0.61)	−0.19 (−0.95, 0.58)
10–14 min	0.18 (−0.79, 1.15)	−0.14 (−1.16, 0.88)
≥ 15 min	0.27 (−0.30, 0.83)	−0.01 (−0.65, 0.62)
Sleep		
Daytime sleep (hours/day)	0.15 (−0.19, 0.32)	0.04 (−0.23, 0.30)
Movement behaviors outside of child care		
Physical activity		
MVPA (hours/day)	−0.03 (−0.39, 0.34)	−0.03 (−0.40, 0.34)
Sedentary behavior		
Screen time (hours/day)	−0.09 (−0.23, 0.06)	−0.07 (−0.22, 0.09)
Non-screen time (hours/day)	−0.01 (−0.20, 0.18)	−0.12 (−0.33, 0.08)
Sleep		
Nighttime sleep (hours/night)	0.22 (0.06, 0.38)*	0.22 (0.05, 0.39)*

β (95 % CI) = unstandardized regression coefficient (95 % confidence interval); *LPA* light physical activity, *MVPA* moderate-vigorous physical activity; Model 1 = accelerometer variables controlled for total wear time; Model 2 = model 1 and variables controlled for age, sex, and parental education; * = *P* < 0.05

## Discussion

This study examined the associations between movement behaviors and BMI z-scores among 19–60 month olds inside and outside of child care, and the potential moderating effects of age, sex, and parental education. Sedentary bouts lasting 1–4 min during child care and nighttime sleep were the only movement behaviors associated with BMI z-score. Demographic factors did not moderate the association between the movement behavior variables and BMI z-score.

To our knowledge, this is the first study to look at the association between sedentary bouts and a health indicator in toddlers and preschoolers. Though the association between sedentary bouts lasting 1–4 min and BMI z-scores was statistically significant in model 1 (*P* = 0.01), it was only borderline non-significant in model 2 (*P* = 0.05). The significant findings of model 1and trend toward significance in model 2 are consistent with two studies in school-age children [26, 27]. There are several potential explanations for an inverse association between short sedentary bouts and BMI z-scores. First, alternating between sedentary and non-sedentary postures requires energy expenditure [28]. The energy expended from repetitive postural transitions may be protective against BMI z-score increases. Second, an important aspect of a healthy physiological system is the ability to adapt to unpredictable stresses and stimuli [29]. Children with a more sporadic movement profile, as a result of frequently changing postures, would present more unpredictable stimuli and stress to the physiological system, which could protect against BMI z-score increases. Conversely, long bouts of sedentary behavior may not offer ideal stimulation for adaptation. Lastly, given the current evidence is cross-sectional and temporality is unknown, it is possible that children with higher BMI z-scores are less likely to engage in shorter sedentary bouts [26]. Longitudinal and experimental studies are needed to further explore the association between sedentary bouts and BMI z-scores both inside and outside of child care in this age group.

The findings for model 1 suggest that for every additional hour/day of sedentary bouts lasting 1–4 min inside child care, there was a 0.8 unit lowering in BMI z-score. Similar findings were seen for model 2, though the model was borderline non-significant. Considering 1.5 h/day remains in sedentary bouts ≥5 min, it would theoretically be possible to decrease BMI z-scores by 1.2 units if all sedentary time was accumulated in 1–4 min bouts. Additionally, more frequent breaks in sedentary time; thus, shorter sedentary bouts, have shown clinically significant health benefits in adults [23, 30, 31]. Therefore, creating habits of short sedentary bouts in young children may contribute to positive health later in life.

Nighttime sleep was positively associated with BMI z-scores in this sample. This is surprising given a negative association between these two variables has consistently been observed in the pediatric literature [2, 32]. It is unclear why this finding was observed. It is thought that one of the key mechanisms for the association between short sleep duration and adiposity indicators in older children is increased food intake [33]. Since this study did not measure food intake, it was not possible to determine the role of food intake in the observed associations. Additionally, given that evidence exists for an association between excess adipose tissue in children and poor sleep quality [34], it could be that children with higher BMI z-scores in this sample required more sleep duration to compensate for lack of quality sleep. Furthermore, a recent meta-analysis found that sleep is positively associated with fat mass in early years children [32]. A high BMI could be reflective of high fat mass, high fat free mass, and/or low height; thus, higher BMI z-scores within this sample may have been driven by high fat mass, with less contribution from fat free mass and height. It is possible that null associations between physical activity and sedentary behaviour measures may also be due to the limitations associated with the height and weight indices used as an adiposity indicator. For instance, previous research has shown inverse associations between more direct measures of adiposity (i.e., dual energy x-ray absorptiometry) and vigorous physical activity in preschoolers [35, 36]. Dual energy x-ray absorptiometry has excellent validity in this age group [37], while weight and height indices have shown to only have good validity for detecting overweight preschoolers [38]. However, BMI has the major advantage of being simple and cost-effective. Therefore, the utility of BMI is undeniable unless a measure is found matching the simplicity, and cost-effectiveness of BMI while also enhancing the validity.

To our knowledge this is the first study to explore the moderating effects of demographic variables on the association between movement behaviors and BMI z-scores. No evidence of moderation was found; thus, the association between movement behaviors and BMI z-scores may be similar across age, sex, and parental education within this age group. In an older sample (3–7 years old), Niederer et al. [39] also found that age did not moderate the association between physical activity and BMI, but no other demographic moderators were explored. Given the limited evidence in this area, and the small sample in the present study, future research is needed to confirm the present findings to understand whether targeted interventions are needed.

There were several limitations within this study, including the use of both objective and subjective measures of movement behaviors. The current study used baseline data collected from a quasi-experimental study, which was primarily concerned with physical activity and sedentary behaviour inside of childcare. As a result, the current study relied on subjective measures for behaviors outside of childcare, and comparisons between behaviors inside and outside of child care could not directly be made. Further, though the majority of subjective measures were adopted from a large national study in Canada [19], the validity and reliability of the measures are unknown. Therefore, the use of parental questionnaires could have introduced measurement error, as these measures are more prone to bias compared to objective measures (i.e., accelerometers). This measurement error is especially true of parental estimates of daytime sleep inside child care. Though, 24 h accelerometer wear protocols are more frequently being used, to our knowledge there is not an algorithm to determine daytime sleep from accelerometer data in this age group without an adequate log-sheet [40]. While accelerometer cut-points were the same as cut-points used in a Canadian national survey [19], they were developed for 1-min epochs and have been validated in preschoolers [41] but not toddlers. Another limitation was that diet may have directly and indirectly influenced BMI z-scores, but it was not measured in this study. Also, since this is a cross-sectional study causal inferences cannot be made. Selection bias and reduced generalizability could have been introduced by a modest participation rate, the excluded participants, and the relatively small sample size. Despite being adequately powered for the primary purpose of the study [42], the relatively small sample size may have also resulted in the study being underpowered to address the secondary objective of the study, which included moderation analyses*.* There are also several strengths such as the age group studied and the inclusion of all movement behaviors across settings. Additional strengths include collecting data from three geographic regions in Alberta, Canada and using objective measures of physical activity and sedentary behavior within child care.

## Conclusions

A negative association between time spent in sedentary bouts lasting 1–4 min and BMI z-scores, and a positive association between nighttime sleep and BMI z-scores were observed in this sample. No other movement behaviors inside or outside of child care were associated with BMI z-scores. Though all children must engage in some sedentary behavior in a day, findings suggest promoting short bouts of sedentary behavior during child care may be important for the primary prevention of overweight and obesity. Future research is needed using large representative samples to confirm these findings.

## Abbreviations

BMI, body mass index; LPA, light-intensity physical activity; MVPA, moderate- to vigorous-intensity physical activity; SD, standard deviation; WHO, World Health Organization

## References

[1] Chaput JP, Carson V, Gray CE, Tremblay MS (2014). Importance of all movement behaviors in a 24 h period for overall health. Int J Environ Res Public Health.

[2] Chen X, Beydoun MA, Wang Y (2008). Is sleep duration associated with childhood obesity? A systematic review and meta-analysis. Obesity.

[3] Janssen I, LeBlanc AG (2010). Review Systematic review of the health benefits of physical activity and fitness in school-aged children and youth. Int J Behav Nutr Phys Act.

[4] Tremblay MS, LeBlanc AG, Kho ME, Saunders TJ, Larouche R, Colley RC, Goldfield G, Gorber SC (2011). Systematic review of sedentary behaviour and health indicators in school-aged children and youth. Int J Behav Nutr Phys Act.

[5] Goldfield GS, Harvey A, Grattan K, Adamo KB (2012). Physical activity promotion in the preschool years: a critical period to intervene. Int J Environ Res Public Health.

[6] Carter PJ, Taylor BJ, Williams SM, Taylor RW. Longitudinal analysis of sleep in relation to BMI and body fat in children: the FLAME study. BMJ. 2011;342. 10.1136/bmj.d2712 PMC3102792 21622518

[7] Tandon P, Grow HM, Couch S, Glanz K, Sallis JF, Frank LD, Saelens BE (2014). Physical and social home environment in relation to children's overall and home-based physical activity and sedentary time. Prev Med.

[8] Couch SC, Glanz K, Zhou C, Sallis JF, Saelens BE (2014). Home food environment in relation to children’s diet quality and weight status. J Acad Nutr Diet.

[9] Duch H, Fisher EM, Ensari I, Harrington A (2013). Screen time use in children under 3 years old: a systematic review of correlates. Int J Behav Nutr Phys Act.

[10] Bushnik T (2006). Child care in Canada.

[11] Geoffroy M-C, Power C, Touchette E, Dubois L, Boivin M, Séguin JR, Tremblay RE, Côté SM (2013). Childcare and overweight or obesity over 10 years of follow-up. J Pediatr.

[12] Carson V, Clark D, Ogden N, Harber V, Kuzik N. Short-term influence of revised provincial accreditation standards on physical activity, sedentary behavior, and weight status in Alberta, Canada Child Care Centers. Early Childhood Educ J. 2015;43(6): 459-65.

[13] Group WMGRS (2006). WHO Child Growth Standards based on length/height, weight and age. Acta Paediatrica (Oslo, Norway: 1992) Supplement.

[14] Onyango AW, De Onis M (2008). WHO child growth standards: training course on child growth assessment.

[15] Cliff DP, Reilly JJ, Okely AD (2009). Methodological considerations in using accelerometers to assess habitual physical activity in children aged 0-5 years. J Sci Med Sport.

[16] Esliger DW, Copeland JL, Barnes JD, Tremblay MS. Standardizing and optimizing the use of accelerometer data for free-living physical activity monitoring. Journal of physical activity & health. 2005;3(1):366-83.

[17] Pate RR, Pfeiffer KA, Trost SG, Ziegler P, Dowda M (2004). Physical activity among children attending preschools. Pediatrics.

[18] Trost SG, Sirard JR, Dowda M, Pfeiffer KA, Pate RR (2003). Physical activity in overweight and nonoverweight preschool children. Int J Obes.

[19] Colley RC, Garriguet D, Adamo KB, Carson V, Janssen I, Timmons BW, Tremblay MS (2013). Physical activity and sedentary behavior during the early years in Canada: a cross-sectional study. Int J Behav Nutr Phys Act.

[20] Janssen X, Cliff D, Reilly J, Hinkley T, Jones R, Batterham M, Ekelund U, Brage S, Okely T: Evaluation of Actical equations and thresholds to predict physical activity intensity in young children. J Sports Sci*.* 2014(ahead-of-print):1–9. 10.1080/02640414.2014.949826 25259944

[21] Altenburg T, de Niet M, Verloigne M, De Bourdeaudhuij I, Androutsos O, Manios Y, Kovacs E, Bringolf-Isler B, Brug J, Chinapaw M (2015). Occurrence and duration of various operational definitions of sedentary bouts and cross-sectional associations with cardiometabolic health indicators: The ENERGY-project. Prev Med.

[22] Carson V, Ridgers ND, Howard BJ, Winkler EAH, Healy GN, Owen N, Dunstan DW, Salmon J (2013). Light-intensity physical activity and cardiometabolic biomarkers in US adolescents. PLoS One.

[23] Carson V, Wong SL, Winkler E, Healy GN, Colley RC, Tremblay MS (2014). Patterns of sedentary time and cardiometabolic risk among Canadian adults. Prev Med.

[24] Willett W, Stampfer MJ (1986). Total energy intake: implications for epidemiologic analyses. Am J Epidemiol.

[25] Heck RH, Thomas SL, Tabata LN. Multilevel and longitudinal modeling with IBM SPSS. Abingdon: Routledge; 2013.

[26] Saunders TJ, Tremblay MS, Mathieu M-È, Henderson M, O’Loughlin J, Tremblay A, Chaput J-P, on behalf of the Qcrg (2013). Associations of sedentary behavior, sedentary bouts and breaks in sedentary time with cardiometabolic risk in children with a family history of obesity. PLoS One.

[27] Carson V, Stone M, Faulkner G (2014). Patterns of sedentary behavior and weight status among children. Pediatr Exerc Sci.

[28] Anan M, Ibara T, Kito N, Shinkoda K (2012). The Clarification of the Strategy during Sit-to-Stand Motion from the Standpoint of Mechanical Energy Transfer. J Phys Ther Sci.

[29] Goldberger AL, Peng CK, Lipsitz LA (2002). What is physiologic complexity and how does it change with aging and disease?. Neurobiol Aging.

[30] Healy GN, Dunstan DW, Salmon J, Cerin E, Shaw JE, Zimmet PZ, Owen N (2008). Breaks in sedentary time: beneficial associations with metabolic risk. Diabetes Care.

[31] Bankoski A, Harris TB, McClain JJ, Brychta RJ, Caserotti P, Chen KY, Berrigan D, Troiano RP, Koster A (2011). Sedentary activity associated with metabolic syndrome independent of physical activity. Diabetes Care.

[32] Ruan H, Xun P, Cai W, He K, Tang Q (2015). Habitual Sleep Duration and Risk of Childhood Obesity: Systematic Review and Dose-response Meta-analysis of Prospective Cohort Studies. Sci Rep.

[33] Hart CN, Carskadon MA, Considine RV, Fava JL, Lawton J, Raynor HA, Jelalian E, Owens J, Wing R (2013). Changes in children’s sleep duration on food intake, weight, and leptin. Pediatrics.

[34] Beebe DW, Lewin D, Zeller M, McCabe M, MacLeod K, Daniels SR, Amin R (2007). Sleep in overweight adolescents: shorter sleep, poorer sleep quality, sleepiness, and sleep-disordered breathing. J Pediatr Psychol.

[35] Janz KF, Levy SM, Burns TL, Torner JC, Willing MC, Warren JJ (2002). Fatness, physical activity, and television viewing in children during the adiposity rebound period: the Iowa bone development study. Prev Med.

[36] Collings PJ, Brage S, Ridgway CL, Harvey NC, Godfrey KM, Inskip HM, Cooper C, Wareham NJ, Ekelund U (2013). Physical activity intensity, sedentary time, and body composition in preschoolers. Am J Clin Nutr.

[37] Karlsson A-K, Kullberg J, Stokland E, Allvin K, Gronowitz E, Svensson P-A, Dahlgren J (2013). Measurements of total and regional body composition in preschool children: a comparison of MRI, DXA, and anthropometric data. Obesity.

[38] Mei Z, Grummer-Strawn LM, Pietrobelli A, Goulding A, Goran MI, Dietz WH (2002). Validity of body mass index compared with other body-composition screening indexes for the assessment of body fatness in children and adolescents. Am J Clin Nutr.

[39] Niederer I, Kriemler S, Zahner L, Bürgi F, Ebenegger V, Marques-Vidal P, Puder JJ (2012). BMI group-related differences in physical fitness and physical activity in preschool-age children. Res Q Exerc Sport.

[40] Barreira TV, Schuna JM, Mire EF, Katzmarzyk PT, Chaput JP, Leduc G, Tudor-Locke C (2015). Identifying children's nocturnal sleep using 24-h waist accelerometry. Med Sci Sports Exerc.

[41] Adolph AL, Puyau MR, Vohra FA, Nicklas TA, Zakeri IF, Butte NF (2012). Validation of uniaxial and triaxial accelerometers for the assessment of physical activity in preschool children. J Phys Act Health.

[42] Cohen J (1992). A power primer. Psychol Bull.

